# Host‐feeding preferences of *Culex pipiens* and its potential significance for flavivirus transmission in the Camargue, France

**DOI:** 10.1111/mve.12802

**Published:** 2025-03-21

**Authors:** Víctor Rodríguez‐Valencia, Marie‐Marie Olive, Gilbert Le Goff, Marine Faisse, Marie Bourel, Grégory L'Ambert, Benjamin Vollot, María José Tolsá‐García, Christophe Paupy, David Roiz

**Affiliations:** ^1^ MIVEGEC, Univ. Montpellier, IRD, CNRS Montpellier France; ^2^ International Joint Laboratory ELDORADO, IRD/UNAM Mexico City Mexico; ^3^ ASTRE, Univ. Montpellier, CIRAD, INRAE Montpellier France; ^4^ Entente interdépartementale pour la démoustication du littoral méditerranéen (EID Méditerranée) Montpellier France

**Keywords:** bloodmeal preferences, Camargue, *Culex pipiens*, forage ratio, France, mosquito‐borne diseases, Usutu, West Nile virus

## Abstract

The spread of the West Nile (WNV) and Usutu (USUV) flaviviruses in Europe in recent decades highlights the urgent need to understand the transmission networks of these pathogens as a basis for effective decision‐making. These viruses are part of a complex disease cycle that involves birds as principal hosts and humans and horses as dead‐end hosts. Our study aims to uncover the intricate relationships between the main mosquito vector of these viruses, *Culex pipiens* L. (Diptera: Culicidae) and its feeding preferences based on the forage ratio among several host species, primarily birds in a land‐use gradient. We estimated the bird host potential to act as a host for flavivirus, the reservoir capacity index, based on forage ratios and potential host competence based on molecular prevalence. We sampled mosquitoes and, at the same time, conducted bird censuses in the Camargue region in southern France, where co‐circulation of these viruses has been reported. Several localities were sampled along a land‐use gradient in peri‐urban, agricultural and natural areas from May to November 2021. We identified 55 vertebrate species in 110 engorged *Cx. pipiens* by PCR amplification and sequencing of mitochondrial 12S and 16S Ribosomal DNA genes. *Culex pipiens* feeds primarily on 51 bird species and secondarily on two mammals, one amphibian and one reptile. Based on forage ratios, we found a preference of *Cx. pipiens* in the Camargue for the order Passeriformes and, more specifically, for *Columba livia domestica* L. (Columbiformes: Columbidae) in agricultural areas, and for *Passer domesticus/montanus* L. (Passeriformes: Passeridae), in agricultural and peri‐urban areas. The natural habitats had significantly higher forage ratio values than agricultural and peri‐urban areas. We suggest that certain key species, such as *Passer* sp., *Columba livia* and *Turdus* sp., might be potentially considered locally relevant hosts for transmission in this area, as they are important for mosquito feeding and also potentially important hosts for flavivirus amplification. These data will be beneficial in understanding host–vector interactions and the relationships between bird communities, mosquito feeding preferences and emerging mosquito‐borne diseases.

## Graphical abstract

- We identified *Culex pipiens* bloodmeals at the species level in natural, agricultural and peri‐urban habitats and calculated host preferences based on forage ratios.
- This analysis showed Passeriformes, and in particular *Columba livia*, *Passer montanus* and *Turdus* sp., to be the preferred *Cx. pipiens* hosts.
- We characterised the potential vector–host–virus networks based on forage ratios and host competence data, and assessed the potential contribution of these two factors to disease transmission.

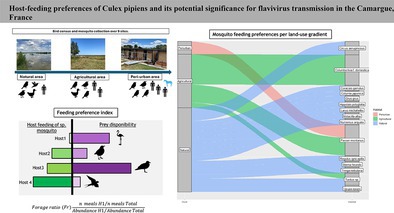

## INTRODUCTION

Vector‐borne diseases pose a significant threat to global public health, with more than 80% of the world's population at risk. Mosquito‐borne diseases represent a significant burden to human and animal health, causing a relevant economic impact (Diagne et al., 2021; Franklinos et al., 2019; Roiz et al., 2024). Vector‐borne diseases are characterised by intricate processes involving interactions between viruses, hosts, vectors, landscape, climate and human activities (Bournez et al., 2019; Huang et al., 2014; Lambin et al., 2010; Reisen, 2010; Tolsá‐García et al., 2023).

*Flaviviruses* are a diverse group of mosquito‐borne RNA viruses that include several human pathogens, such as the dengue, Zika and yellow fever viruses, as well as the West Nile (WNV) and Usutu viruses (USUV), which belong to the Japanese encephalitis serocomplex (Tolsá‐García et al., 2023). WNV and USUV are maintained in an enzootic cycle between *Culex* mosquitoes and birds (that varied on host competence at a species level) but can also infect other vertebrates, such as horses and humans, which are considered dead‐end hosts (Wang et al., 2020). The role of most wild mammals as potential amplifying hosts for WNV is, to date, uncertain, as experimental infections have resulted in no or low‐level viremia (Root & Bosco‐Lauth, 2019). Despite vaccines being available for horses, no specific treatments or vaccines for WNV or USUV are available for humans, highlighting the importance of proper viral prevention and vector control (Angeloni et al., 2023; Constant et al., 2020).

On multiple occasions over many decades, WNV has entered southern Europe and the Mediterranean Basin, including the Camargue region in the south of France, where the first WNV outbreak in France was reported in 1962 (Murgue et al., 2001). The region's unique ecosystem, characterised by wetlands and diverse avian species, provides an ideal environment for amplifying and transmitting this agent. USUV was first documented in the Camargue region in 2015 (Eiden et al., 2018). However, it has been in circulation in the south of France since 2009, which means there is a long‐term establishment and enzootic circulation in this ecologically rich area (Bournez et al., 2019; Cailly et al., 2011; Constant et al., 2020). The endemic enzootic co‐circulation of WNV and USUV has implications for public health and highlights the importance of studying the dynamics of these pathogens in the Camargue (Bahoun et al., 2016; Pacenti et al., 2020; Vahey et al., 2021; Zeller & Schuffenecker, 2004). A crucial aspect of these dynamics is the presence of competent birds and disease‐transmitting vectors, a role played primarily by the mosquito species *Culex pipiens* and secondarily by *Culex modestus* Ficalbi (Diptera: Culicidae) (Eiden et al., 2018; Soto et al., 2023; Tolsá‐García et al., 2023). The seasonal dynamics of *Culex pipiens* L. (Diptera: Culicidae) and *Cx. modestus*, which are associated with temperature, rainfall and water management patterns, have been characterised for the Camargue and other Mediterranean wetlands, being present from June to September with population peaks that varied among localities (Balenghien et al., 2006; Ponçon et al., 2007; Roiz et al., 2015).

Understanding the complex interplay between the vector and the host viral reservoirs in order to mitigate the impact of flaviviruses requires a comprehensive, integrated approach at the local level (Lambin et al., 2010; Reisen, 2010; Roiz et al., 2019). A significant factor conditioning arboviral emergence and transmission is the vector–host interaction. Mosquito feeding patterns are studied by analysing the bloodmeals of females to identify their origin and describing the hosts that mosquitoes feed on in nature. Several factors are known to influence host‐feeding patterns: the mosquito's innate preference for particular hosts, host availability, host defences and mosquito host preference (Clements, 1999; Fikrig & Harrington, 2021). Host preferences describe mosquito species' tendency to select certain hosts over others. They are studied by analysing these patterns in relation to host availability with feeding metrics like the forage ratio or in host choice experiments (Fikrig & Harrington, 2021).

We focussed on *Cx. pipiens*, the main vector species ensuring transmission of WNV and USUV in Europe based on multiple studies (Angeloni et al., 2023; Tolsá‐García et al., 2023). The analyses of bloodmeal patterns in different areas of the world have shown the opportunistic behaviour of this species, which is related to its wide geographical heterogeneity and which has recently been confirmed by a global meta‐analysis of host‐feeding patterns (Apperson et al., 2002; Molaei & Andreadis, 2006; Wehmeyer et al., 2024). One third of the bloodmeals of *Cx. pipiens* have been found to come from human, avian and non‐human mammalian hosts, with only a few from amphibians and reptiles, which is due to several factors, such as host availability (Wehmeyer et al., 2024).

Using field data, we aim to focus on providing an overview of the complex interactions between *Cx. pipiens* and its host vertebrate species, mainly birds, in the Camargue according to habitats and seasons. We relate these preferences to the reservoir capacity index of each bird species for WNV based on molecular prevalence, in order to highlight those interactions that could be useful in determining the niche of transmission of viral ecological cycles in Mediterranean wetlands.

## METHODS

### Study area and experimental design

This study was conducted in the Camargue region, a wetland area in the south of France. At the delta of the Rhône River, the Camargue is one of the largest and most biodiverse wetlands in the Mediterranean and is the habitat of more than 300 sedentary or migratory bird species (Lebarbenchon et al., 2010). We sampled mosquitoes and took bird censuses between May and November 2021 across a land‐use gradient ranging from peri‐urban (artificial surfaces) to agricultural (rice fields and crops) and natural areas (marshes and forest), which are referred to as different habitats.

**FIGURE 1:**
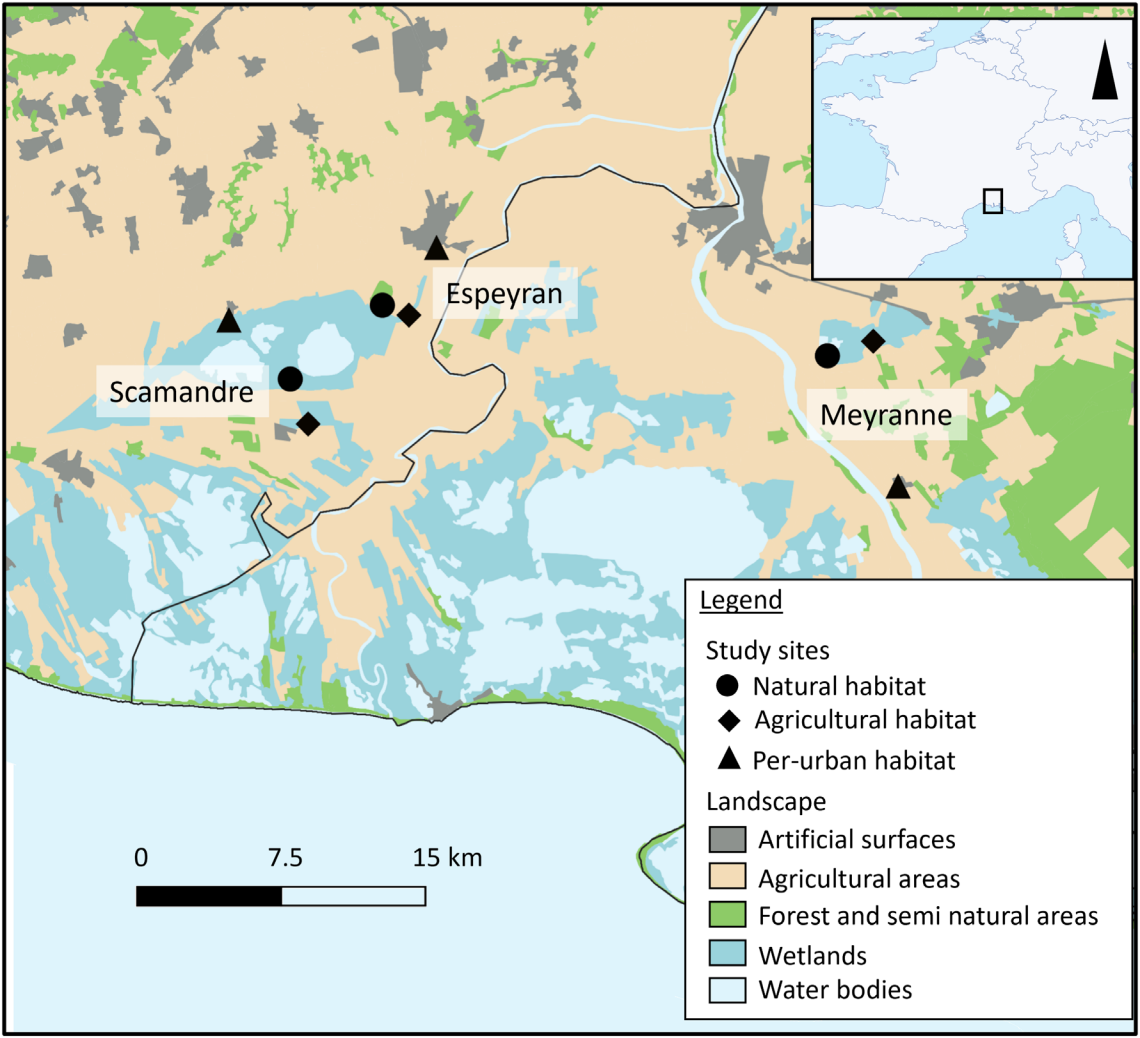
Map showing the three study areas in the Camargue region with their land‐use classifications. Each study area incorporates the three land‐use types (habitats), each of which encompasses different types of land cover: peri‐urban (black triangles; artificial surfaces), agricultural (black diamonds; agricultural areas) and natural (black dots; wetlands and forests).

Three localities of each of the three habitats were chosen for each area, and overall, nine sampling locations were selected among three study areas, with three replicates per habitat. Two of the study areas were located in the Gard department, Scamandre (SCA) and Espeyran (ESP), with three mosquito trapping locations, respectively, and one was located in the Bouches‐du‐Rhône department, Meyranne (MEY), with three mosquito trapping locations (Figure 1). Measures introduced to limit the transmission of SARS‐Cov2 delayed the fieldwork during the spring and created logistical limitations, which hampered continuous fieldwork. Table S1 presents a landscape characterisation of each study site based on the proportions of each land cover type in a 600 m radius buffer zone extracted from the CORINE Land Cover1 geographical database.

### Mosquito sampling

Adult mosquitoes were captured using Biogent ‘BG‐Sentinel 2’ traps (BGS) baited with CO_2_ and a BG‐lure supplied with a 12 V battery. Mosquitoes were captured over 24 h once every 3–4 weeks at each study site alternately. In addition to the capture of blood‐fed females, mosquitoes were collected during the morning (8–10 AM) in collection cups inside two resting traps (i.e., 170‐L pop‐up garden bags) (Sauer et al., 2020) placed in each sampling site at a distance of at least 100 m from the BGS, one positioned slightly above the ground (30–50 cm), the other at about 1.5 m from the ground. Resting females were aspirated from the pop‐ups for two consecutive days per week using a Prokopack® aspirator model 1419 (John W. Hock Company), while additional opportunistic sampling was carried out by aspirating on vegetation in two sessions of 10 min each per locality per week (Maia et al., 2011).

### Storage and identification of mosquitoes

At the end of each collection session, the catch bags and collection cups were labelled and stored in an ice chest until they were sent to the laboratory the same day, where they were immediately placed in freezers at −20°C until identification the same week. The mosquitoes were then morphologically identified and grouped by sex and species using taxonomic identification keys (Becker et al., 2010). The digestion stage of the blood‐fed mosquitoes was visually determined based on the Sella scale; these blood‐fed mosquitoes were then stored individually (Detinova et al., 1962). All mosquitoes were maintained at −20°C until molecular identification of bloodmeals.

### Bloodmeal identification

We focussed on identifying the bloodmeals of the species *Cx. pipiens*, this being the objective of the study. The abdomens of the blood‐fed females were individually ground using a TissueLyser® and metal beads for three 1‐min cycles at 30 Hz, with a pause of 1 min between each cycle to prevent heating. DNA was extracted using a protocol based on CTAB (Morlais et al., 2004). DNA pellets were resuspended in 30 μL of nuclease‐free water before being quantified by Nanodrop. The DNA extracted was used as a PCR template with primers designed to amplify 12S and 16S sequences of vertebrate species preferentially. Primers were selected to obtain the best results with the fewest redundancies; although both primers were designed for vertebrate identification, 16S was better at amplifying mammal sequences, while 12S was better at bird sequences. The sequences of the primers were 5′‐CTGGGATTAGATACCCCACTAT‐3′ and 5′‐GTCGATTATAGGACAGGTTCCTCTA‐3′ for 12S, and 5′‐CGGTTGGGGTGACCTCGGA‐3′ and 5′‐GCTGTTATCCCTAGGGTAACT‐3′ for 16S (Goldberg et al., 2009). PCR amplifications were carried out in a volume of 25 μL containing 0.2 μM of each primer, 1 μM of each blocking primer, 200 μM dNTP (with dUTP replacing dTTP), 0.3 U AmpErase® Uracil N‐glycosylase (Invitrogen, Carlsbad, CA, USA), 4 mM MgCl2, 1X PCR buffer, 1.25 U Platinum® Taq polymerase and 2.5 μL of DNA template (200–600 ng/μL). Cycling conditions consisted of an initial denaturation step at 95°C for 5 min, followed by 50 cycles at 94°C for 30 s, 55°C for 30 s, 72°C for 1 min and a final extension at 72°C for 10 min. The PCR of the purified products of both strands was directly sequenced by Macrogen®. Geneious (version 1.6) was used to visualise, trim and clean the sequences and obtain consensus sequences from forward and reverse sequences. Consensus sequences were used to search correspondences using BLAST® (https://blast.ncbi.nlm.nih.gov/Blast.cgi). Hosts were identified to the species level with a query coverage of at least 95% and a matched identity of 95% (Ashfaq et al., 2014).

### Bird census

To assess the relative abundance and richness (number of species) of avian hosts, a professional ornithologist (B.V.) carried out structured bird censuses at each of the three habitats in each of the three study areas three times (27 in total) during the mosquito season in July, September and October 2021. Point counts were performed for 6 min at each mosquito trapping location (one at the trap itself and the other four at 150–200 m in each cardinal direction). The counts were made from early morning until 4 h after sunrise within 3 days of mosquito trapping. Only the visual and auditory contacts in front of the observer were counted, as the area behind was disturbed while they were walking. For each contact, the following variables were recorded: species, number of individuals, distance and altitude at the moment of the record. Any individuals not observed during the counts were recorded as ‘free observations’ (Sutherland et al., 2004).

### Forage ratio

The forage ratio (FR), which quantifies the vector's selection of a particular vertebrate host, was calculated using the following formula:

$$\text{Forage ratio}\;\left(\mathrm{Fr}\right)\frac{n\;\text{meals}\;H1/n\;\text{meals Total}}{\text{Abundance}\;H1/\text{Abundance Total}}.$$

where *n* meals H1 = Number of blood‐fed samples obtained for Host 1. *n* meal Total = Total amount of blood‐fed samples obtained for all hosts in the area. Abundance H1 = Abundance obtained in the census for Host 1. Abundance Total = Total abundance obtained in the census for all hosts in the area.

An FR of 1.0 indicates neutral behaviour, neither selecting nor avoiding a given host; an FR significantly >1.0 indicates selective behaviour, while a value <1.0 indicates the avoidance of a given host in favour of a different one in the area. Where a vertebrate species was found to be a bloodmeal host but was not reported in the vertebrate surveys, it was assigned an abundance equal to the lowest observed vertebrate abundance in the locality (Goldberg et al., 2009; Gunathilaka et al., 2016). Where a vertebrate species was observed in the census but not as a bloodmeal host, it was given a value equal to the minimum per locality, one bloodmeal observation.

### Data analysis

We built a database by merging the results of the bird censuses, the mosquito captures and the feeding results. To analyse the impact of seasonality, we decided to group the bloodmeal identification data into three periods (season) related to the census dates: (1) early summer, from May to July; (2) late summer, August and September; and (3) early autumn, October and November. We calculated the abundance (numbers) and richness (number of species) of birds and mosquitoes, and the forage ratios. To assess the reservoir capacity index that each bird may pose for each habitat based on the feeding preferences for this area, we multiplied the FR by the estimated host competence of each bird species for WNV based on molecular prevalence (Komar et al., 2018; Tolsá‐García et al., 2018), and we divided by 100 to calculate a percentage. As there were insufficient host competence data for Usutu, we assumed it to be commensurate with West Nile, based on Nikolay (2015), which suggested that their transmission overlaps substantially in Europe in similar bird populations. We developed a Generalised Linear Model with a negative binomial distribution (as the data were over‐dispersed) to investigate whether the forage ratio was influenced by habitat (land uses) and season. We also investigated the effect of habitat on host richness and abundance. Statistical and graphical analysis was carried out in R version 3.3.0 (R Core Team, 2021).

## RESULTS

### Mosquito capture

In total, 39,631 mosquitoes, 36,163 females (4186 engorged or gravid) and 3468 males, were captured between May and November 2021 (Table S2). Fifteen mosquito species were identified, with *Cx. pipiens* (38.3%) the most abundant species in all the land‐use types, with 14,389 individuals, of which 13,831 were unfed females, 140 were blood‐fed females and 418 were males. This species was followed by *Aedes caspius* Pallas (Diptera: Culicidae) (35.7%), which was surpassed in only one location (ESP03) by a mosquito species belonging to the *Anopheles maculipennis* Meigen (Diptera: Culicidae), complex. The abundance and richness of species within our land‐use gradient are illustrated in Figure S1, panel A. The agricultural areas had the highest number of species (14) and the highest mosquito abundance (14,781). There were slightly fewer species in the urban (12) and natural (13) areas, with around 10,500 mosquitoes in each.

### Bird census

A total of 11,221 individuals belonging to 130 species and 48 avian families were identified (Table S3). The agricultural habitat was the land‐use type with the greatest abundance of birds, with a total of 3379 individuals of 104 species, the most frequent being *Carduelis carduelis* Brisson (Passeriformes: Fringillidae), *Ichthyaetus melanocephalus* Temminck (Charadriiformes: Laridae) and *Plegadis falcinellus* L. (Pelecaniformes: Threskiornithidae) (Figure S1, panel B). Fewer birds were counted in the natural habitat (3166), but the greatest number of species (113), the most frequent being *Fulica atra*, L. (Gruiformes: Rallidae), *Motacilla flava* L. (Passeriformes: Motacilidae) and *Columba palumbus*. L. (Columbiformes: Columbidae). Of the three land‐use areas surveyed in the census, the peri‐urban habitat had the lowest abundance of birds (1302) and the least species richness (75) (Figure S1, Table S3). The bird richness significantly varied in a gradient from natural to more anthropic habitats, being significantly higher in natural to agricultural areas, and in agricultural areas to peri‐urban habitats (Figure 4, Table 2).

### Bloodmeal identification

We tested all 140 blood‐fed *Cx. pipiens* females that we were able to collect; we identified the bloodmeal source at the host‐species level in 110 samples, while the remaining samples were of insufficient quality for analysis. Our results showed that most of them feed primarily on birds and mammals. *Columba livia* L. (Columbiformes: Columbidae) and *Pica pica* L. (Passeriformes: Corvidae), were the most common host species identified. A total of 31 different host species were identified, of which 27 were birds, two were mammals (*Vulpes vulpes L. (Carnivora: Canidae) Bos taurus L. (Artiodactyla: Bovidae)*), one was an amphibian (*Hyla meridionalis, Boettger (Anura: Hylidae*) and one was a reptile (*Lacerta lepida Daudin,(Squamata: Lacetidae)* species (Table 1).

**TABLE 1:** Vertebrate host species and numbers of individuals identified from molecular analysis of DNA extracted from blood‐fed specimens collected in southern France, 2021.

Order	Vertebrate host species identified	Individuals (*n*)
Birds	*Alectoris rufa*	4
	*Ardea cinerea*	2
	*Calliope calliope*	2
	*Cataponera turdoides*	1
	*Chloris chloris*	2
	*Circus cyaneus*	2
	*Columba livia f. domestica*	21
	*Copsychus sechellarum*	1
	*Coracias garrulus*	9
	*Coturnix japonica*	2
	*Emberiza schoeniclus*	1
	*Fringilla montifringilla*	2
	*Gallinula chloropus*	1
	*Garrulus glandarius*	3
	*Loxops coccineus*	2
	*Numida meleagris*	1
	*Nycticorax nycticorax*	3
	*Parus major*	1
	*Passer montanus*	24
	*Pavo cristatus*	1
	*Phylloscopus trochiloides*	1
	*Pica pica*	6
	*Staphida castaniceps*	1
	*Streptopelia decaocto*	2
	*Turdus philomelos*	2
	*Turdus* sp.	6
	*Upupa epops*	1
Mammals	*Bos taurus*	3
	*Vulpes vulpes*	1
Reptiles	*Lacerta lepida*	2
Amphibians	*Hyla meridionalis*	1
Total		110

### Forage ratio

**FIGURE 2:**
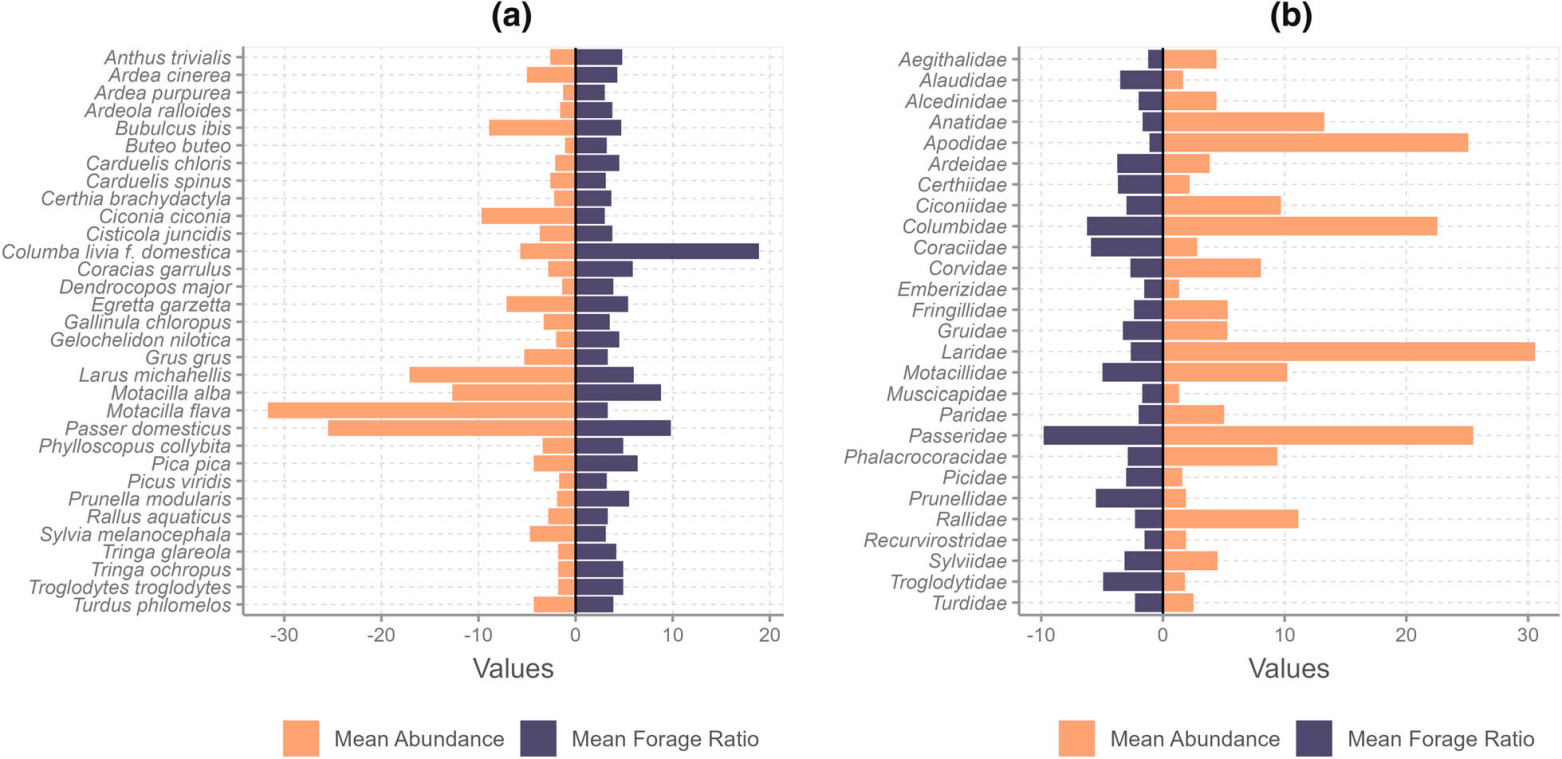
Average forage ratio (selectivity index) of *Culex pipiens* for the vertebrate families (left panel: a) and the principal host species (right panel: b) compared with the mean host abundance. The forage ratio values are represented on the right side of the *y*‐axis; the higher the value, the greater the selectivity of *Cx. pipiens* for the host. The values on the left side of the *y*‐axis represent the mean abundances of each bird family (panel a) and each host species (panel b).

**FIGURE 3:**
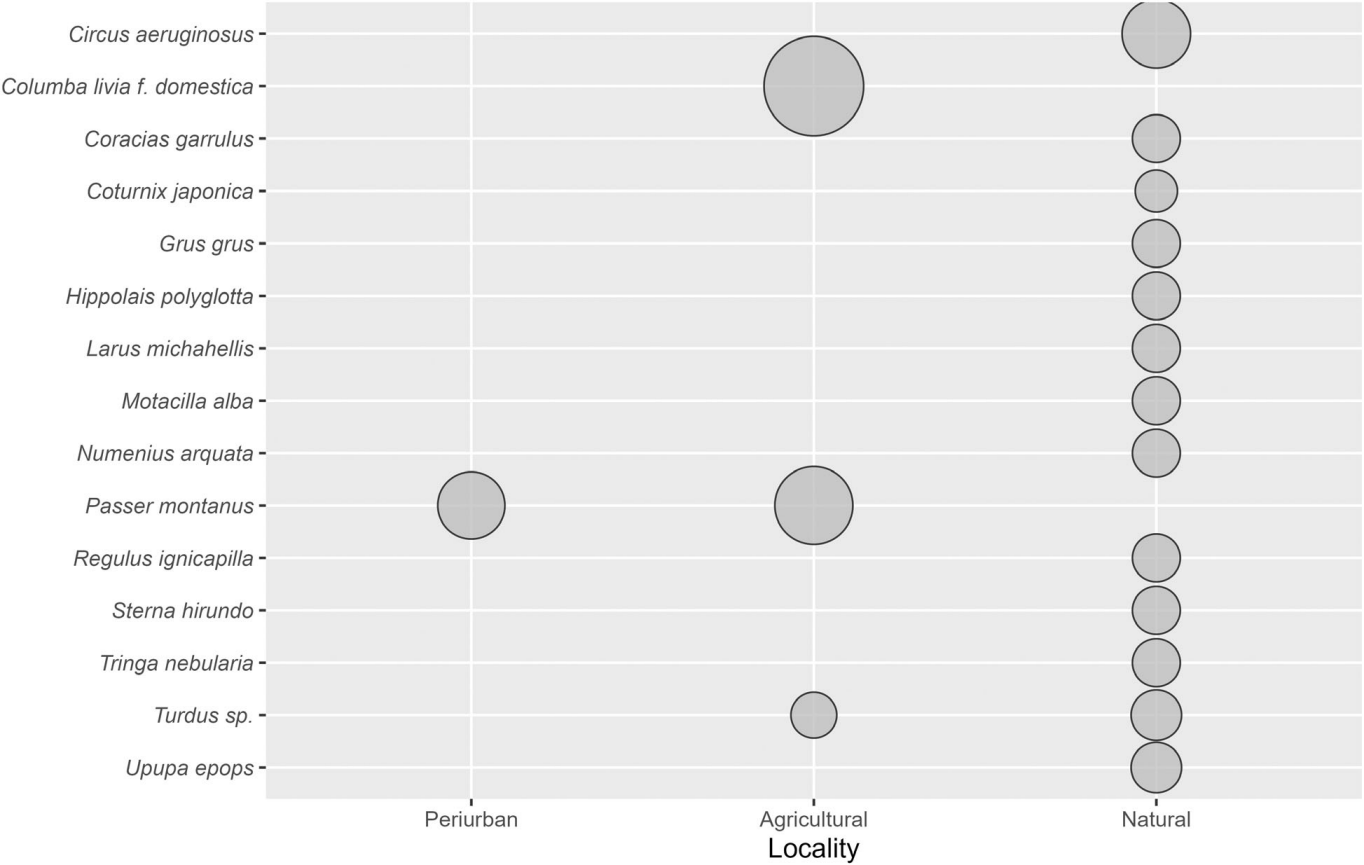
Host preferences based on *Culex pipiens* forage ratios. Results for the principal bird species in the different habitats: peri‐urban, agricultural and natural.

Forage ratios were estimated based on the values calculated for the identified bloodmeals. A comparison of the bird abundances with mosquito selectivity in each locality revealed no clear relationship between the number of individuals of each bird species and the mosquitoes' preferences for a particular bird order or species (Figure 2). Passeriformes is *Cx. pipiens*' preferred order (Figure S2), with a mean FR of 6.3. The forage ratios were highest in the Columbidae (mean FR = 6.2) and Passeridae (mean FR = 9.8) families, and secondarily in the Accipitridae, Gruidae, Laridae, Motacilidae, Sylvidae and Turdidae families (Figure 2a). *Culex pipiens* preferred the following vertebrate species in our study: *Columba livia f. domestica, Gmelin (Columbiformes: Columbidae), Passser domesticus/montanus, L. (Passeriformes: Passeridae), Circus aeruginosus, L. (Accipitriformes: Accipitridae), Turdus* sp., *Motacilla alba L. (Passeriformes:Motacillidae), Upupa epops, L. (Bucerotiformes: Upupidae), P. pica, Tringa nebularia Gunnerus (Charadriiformes: Scolopacidae), Milvus migrans Boddaert, (Accipitriformes: Accipitridae) , Coracias garrulus L. (Coraciiformes: Coraciidae), Larus michahellis, Naumann (Charadriiformes: Laridae), Serinus serinus L. (Passeriformes: Fringillidae), Phylloscopus trochilus L. (Passeriformes: Phylloscopidae) and Hippopolais polyglota* Vieellot (Passeriformes: Acrocephalidae), among others (mean FR = 3.1–18.2) (Figures S3 and S4). *Culex pipiens* had a preference for *Columba livia domestica* (particularly in agricultural areas) and *Passer domesticus/montanus* L. (Passeriformes: Passeridae) in agricultural and peri‐urban areas of the Camargue, and a secondary preference for several other species (Figure 3).

There was greater heterogeneity in *Cx. pipiens*' host preferences in the natural habitat (Figure 3). The number of species selected by *Cx. pipiens* (defined as having a mean forage ratio higher than 20) decreases following a gradient of anthropic perturbation. In natural areas, 13 species were selected (*Circus aeruginosus, Coracias garrulus, Coturnix japonica, Temminck y Schlegel, (Galliformes: Phasianidae), Grus Grus, L., (Gruiformes: Gruidae), Hippopolais polyglota, Larus michahellis, Motacilla alba, Numenius arquata, L. (Charadriiformes: Scolopacidae), Regulus ignicapilla, Temminck (Passeriformes: Regulidae), Sterna hirundo, L. (Charadriiformes: Laridae), Tringa nebularia, Turdus* sp. and *Upupa epops*). In agricultural areas, the selectivity is reduced to 3 (*Columba livia, Passer domesticus, Turdus* sp.), attaining one species in peri‐urban areas (*Passer domesticus/montanus*).

**FIGURE 4:**
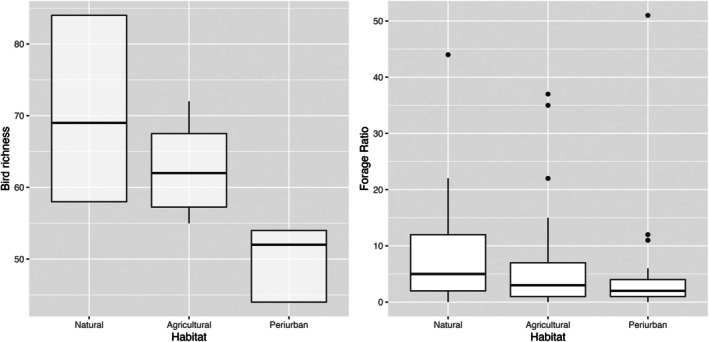
Relationship among habitat and bird richness (left panel), and habitat and forage ratio (right panel). These differences among habitats are statistically significant (Table 2).

The mosquitoes appear to avoid a wide range of bird species (Figure S5). Analysis of selectivity (forage ratios) across the seasons showed a clear seasonal shift concerning several species. For example, the forage ratio of *Co. livia* diminished in natural and agricultural areas in late summer but increased in early autumn. The FR of *Passer domesticus/montanus* increased in late summer in peri‐urban and agricultural areas (Figure S6). The forage ratio is significantly reduced in a gradient from less to more anthropic habitats, being significantly higher in natural than in agricultural areas, in natural than in peri‐urban and in agricultural than in peri‐urban habitats (Figure 4, Table 2). We did not detect statistical differences in the forage ratio among seasons (Table S4, Figure S7), hypothesising that it might be more species‐specific (Figure S6). We found that the number of bloodmeals on a particular host does not have a significant relationship with the abundance of that host (Table S4, Figure S7). We detected differences in host abundance among habitats, with natural and agricultural habitats having statistically higher values than peri‐urban (Table S4, Figure S7).

**TABLE 2:** Model estimates for the GLM negative binomial for the effect of habitat on bird richness and the effect of habitat on forage ratio.

Dependent and independent variables	Values	Estimate	Standard error	*Z* value	*p*‐value
Effect of the habitat on bird richness	Agricultural vs. natural	−0.114	0.007	−14.76	<0.0001
	Agricultural vs. peri‐urban	0.227	0.008	26.59	<0.0001
	Natural vs. peri‐urban	0.341	0.0008	38.60	<0.0001
Effect of the habitat on forage ratio	Agricultural vs. natural	−0.33	0.07	−4.55	<0.0001
	Agricultural vs. peri‐urban	0.54	0.08	6.12	<0.0001
	Natural vs. peri‐urban	0.87	0.09	9.74	<0.0001

*Note*: Model estimates include a post hoc contrast test (as the response variable is a categorical variable). Related graphs are in Figure 4. The explained deviance of the effect of the habitat on richness is 54.6% and the explained deviance of the effect of habitat on forage ratio is 7.5%.

**FIGURE 5:**
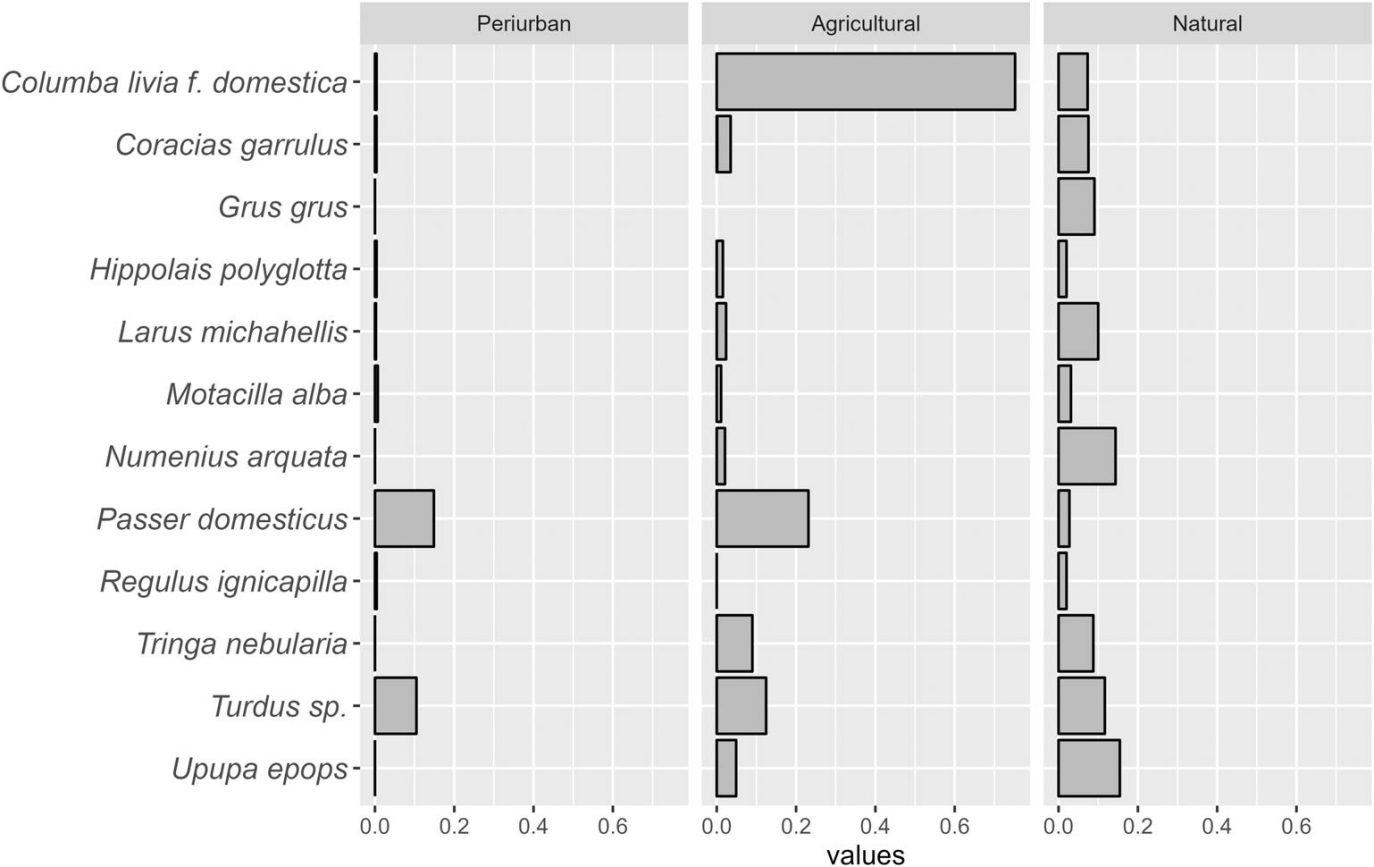
West Nile virus (WNV) reservoir capacity index for the main bird species. We multiplied the forage ratio by the estimated host competence of each bird species for WNV based on molecular prevalence (Tolsá‐García et al., 2018), and we divided by 100 to calculate a percentage.

In natural habitats, *Cx. pipiens* has a preference for a range of multiple bird species with varied potential reservoir capacity index (Figure 5). With regard to the more human impacted habitats, the agricultural and peri‐urban areas, we observe that fewer host species with a varied potential reservoir capacity are being selected (Figure 5). *Turdus* sp. and *Passer* sp. are frequently bitten and have, in general, a high reservoir capacity, also inside Passeriformes (Figure S8). Thus, they have a higher possibility of acting as relevant hosts for WNV and USUV in Camargue (Figure 5).

## DISCUSSION

In our study, we focussed on one of the crucial underlying mechanisms for arbovirus maintenance and emergence: the host preferences (forage ratio) of a competent flavivirus vector, *Cx. pipiens*, in a land‐use (habitat) gradient. We found a wide heterogeneity in the host preferences of *Cx. pipiens* in natural habitats, while in more anthropic habitats, its preferences were for fewer bird species, particularly *Passer domesticus/montanus* in peri‐urban and agricultural habitats and *Co. livia* and *Turdus* sp. in agricultural habitats. Natural habitats have significantly higher forage ratio values than agricultural and peri‐urban habitats, being also the ones that host the higher bird richness (Figure 4, Table 2). We also observed that these patterns varied according to the season, but for a particular species, confirming previous research (Janousek et al., 2014). However, the differences in forage ratio among seasons in our study were not statistically significant. We suggest that certain key species, such as *Passer* domesticus/*montanus, Co. livia* and *Turdus* sp., might be important hosts for mosquito feeding. As they are also potentially important hosts for flavivirus amplification, they might be potentially considered locally relevant actors for the transmission in this area (Kilpatrick et al., 2006).

Passeriformes is the most frequently bitten (but also the most abundant) vertebrate order, especially members of the Passeridae, Motacilidae and Turdidae families. Within the Passeriformes order, *Cx. pipiens*' preferred blood is from *Passer domesticus/montanus, Turdus* spp., *Motacilla alba, P. pica* and *Serinus serinus*. Particular Passeriformes species, such as *Passer domesticus/montanus*, and particular families, such as Sturnidae, may play a role in flavivirus amplification as they have been shown to have high host competence for WNV and USUV (Nikolay, 2015; Pérez‐Ramírez et al., 2014). Although host competence is species‐specific, the forage ratio patterns in each habitat and locality and in each season may determine changes in transmission risk. In a similar biogeographic environment study in Doñana in the south of Spain, we suggested that Passeriformes play a vital role in WNV and USUV amplification (Roiz et al., 2019). *Passer domesticus/montanus* together with *Turdus* species are very frequently bitten and highly competent, and are hence potential key hosts (Pérez‐Ramírez et al., 2014; Tolsá‐García et al., 2018). Our results also show that the family Columbidae and the species *Columba livia* are frequently bitten, potentially being optimal sentinel species for prevalence studies among habitats. Other abundant Columbiformes species, such as *Streptopelia decaocto*, L. Frivaldszky (Columbiformes: Columbidae) or Sturnidae species, such as *Sturnus vulgaris*, or relevant species such as *P. pica*, were avoided in our study, despite being identified as hosts in other studies (Jourdain et al., 2007). Therefore, further studies are needed to confirm or refute these results. Nonetheless, these are important groups, as several other species and orders might also be, depending on location and season.

Bird samples taken in 2019 (Constant et al., 2020) revealed the presence of WNV RNA in the domestic pigeon (*Co. livia*) and the song thrush (*Turdus philomelos, Brehm (Passeriformes: Turdidae*), two relevant species that were selected in our study. On the other hand, in the same study, WNV RNA was also detected in the Eurasian collared dove (*S. decaocto*) and in the Eurasian sparrowhawk (*Accipiter nisus, L. (Accipitriformes: Accipitridae*) species that were not selected in our study, although another Accipitridae, *Circus aeruginosus*, was a preferred host in our study.

This heterogeneity in host selection may be a crucial factor in reducing or increasing the risk of disease transmission, which also depends on the composition of the population (more or fewer competent hosts) and on bird richness. In our study, we found a tendency to select fewer hosts, particularly with high reservoir capacity and abundances (mostly Passeriform species), as the bird richness of the habitats decreased from natural to peri‐urban, having statistically lower values of richness and forage ratios.

In turn, host bird abundance, although an important factor, does not have a significant relationship with selection (forage ratio) and, therefore, seems not to be a determining factor when studied in all habitats and seasons. The composition and richness of the bird communities and the relative abundances seem to have greater weight, potentially determining the changes in the forage ratio among habitats.

The limit of this study is the number of captured *Cx. pipiens* fed females in the field, being a relevant constraint of investigating mosquito–host interactions, the fact that capturing bloodmeal mosquitoes and successfully amplifying the DNA in the bloodmeals is a complex and labour‐intensive process (Estep et al., 2012). To successfully collect a higher number of *Culex* blood‐fed females, it remains critical to further elucidate the resting behaviour of the local population of resting females of the particular species, and to locally adapted appropriate sampling methods after testing multiple options to increase the efficacy of the fieldwork. In our study, we captured and identified a percentage of the bloodmeals at the species level only for *Cx. pipiens*, which is the focus of our study, and we applied a conservative assumption to estimate the information gaps. In particular, we assumed a minimal abundance for birds not reported in the census but detected from bloodmeals and a minimal number of bloodmeals for vertebrate hosts detected in the census but not from the bloodmeals. These conservative assumptions helped us to identify species that may potentially serve as amplifying hosts. In relation to the statistical analysis, for the statistically significant model of the effect of habitat on forage ratio, we found a moderate explained deviance of 7.5%. Therefore, the results should be treated with caution and confirmed with more exhaustive bloodmeal sampling and identification, although this will need a more intensive longitudinal work in the field for addressing local resting habitats and appropriate locally adapted sampling methods.

Following other authors, we used bird abundances to calculate the FR (Chaves et al., 2010; Estep et al., 2012; Komar et al., 2018). Goldberg et al. (2009), on the other hand, used host density to calculate the FR, which is a more accurate method, as it considers that host's home range. However, certain assumptions must be made, such as that the ornithologist's visual range is always equal to the home range. On the whole, we obtained consistent results from abundances, in line with other authors (Gunathilaka et al., 2016).

In future studies, it would be important to differentiate between the two *Cx. pipiens* biotypes: *Cx. pipiens pipiens* (considered to be strictly ornithophilic) and *Cx. pipiens molestus* (considered anthropophilic/mamophilic) as they may have different host‐feeding patterns (Haba & McBride, 2022). However, there is frequent hybridisation between the two biotypes in the Mediterranean basin, resulting in mixed feeding patterns and a wide range of hosts on which *Cx. pipiens* feeds (Shaikevich et al., 2016). Another important issue is to clarify the role of the secondary vector *Cx. modestus* in the Camargue, as, despite particular efforts, insufficient numbers of engorged females of this species were captured in the field to allow for any speculation in this regard. Indeed, this species had a low abundance in the localities studied except for a single capture site (MEY1), where there was a moderate density in 2021. This species has particular peaks of abundance and a specific spatiotemporal niche in semi‐permanent waters, rice fields and irrigation canals, as observed in the Camargue and other Mediterranean areas (Ponçon et al., 2007; Roiz et al., 2015; Soto et al., 2023). It has been suggested that *Cx. modestus* is important for the enzootic transmission cycle, but is significantly affected by local anthropogenic changes (Cailly et al., 2011; Ponçon et al., 2007). More thorough studies are needed, as it is not clear whether the role of this species in the Camargue has been underestimated or overestimated. Automated capture techniques may be considered in the future, as well as new protocols that would allow amplicon‐based metabarcoding (L'Ambert et al., 2023). This would improve our chances of accurately identifying multiple hosts of several bloodmeals from one mosquito. An important caveat relates to the data on host competence. For Usutu virus, these data are limited and it is therefore assumed to have a similar cycle to WNV (Nikolay, 2015). Furthermore, there is little information on West Nile in Europe (Pérez‐Ramírez et al., 2014), and we have to rely on data from North America (Tolsá‐García et al., 2018). These variations in host competences are a significant limitation to accurately assessing transmission risks, and for the moment the information is incomplete.

Our view is that the network of vector–host interactions may add useful data to further define the underlying mechanisms of the relationship between habitat types and disease risk. These types of data with several vectors might allow us to characterise the potential vector–host–virus networks and their contribution to disease transmission (Santiago‐Alarcon, 2022; Tolsá‐García et al., 2025) and to demonstrate if anthropic perturbation might reduce connectivity in host–vector networks. These results might be useful for characterising the niche of transmission and designing surveillance strategies and targeted vector control campaigns using, together with *Cx. pipiens*, these bird species as sentinels or indicators.

Our results confirm the previously described opportunistic behaviour of *Cx. pipiens* (Griep et al., 2025; Wehmeyer et al., 2024), since its host selectivity seems to vary and encompasses different species in the habitat gradient. Based on our results, we speculate that, due to the opportunistic behaviour of *Cx. pipiens*, the reduction in bird richness in agricultural and peri‐urban areas can increase the risk of virus transmission by increasing the biting rate of vector mosquitoes for particular species (such as *Passer, Turdus or Columba*) that will act as relevant WNV hosts, therefore increasing the risk of enzootic transmission. A hypothesis resulting from this study is the question of whether conserving the habitats in the Camargue and preserving bird richness will potentially protect against the emergence of different flaviviruses, such as the West Nile and Usutu viruses.

## AUTHOR CONTRIBUTIONS

**Víctor Rodríguez‐Valencia:** Investigation; writing – original draft; validation; writing – review and editing; formal analysis. **Marie‐Marie Olive:** Conceptualization; investigation; methodology; project administration; supervision; data curation; writing – original draft; writing – review and editing. **Gilbert Le Goff:** Conceptualization; investigation; methodology; project administration; data curation; writing – original draft; writing – review and editing. **Marine Faisse:** Investigation; data curation; validation; writing – review and editing. **Marie Bourel:** Investigation; writing – review and editing. **Grégory L'Ambert:** Investigation; writing – review and editing. **Benjamin Vollot:** Investigation; writing – review and editing. **María José Tolsá‐García:** Writing – review and editing; formal analysis. **Christophe Paupy:** Writing – review and editing; investigation; methodology. **David Roiz:** Conceptualization; writing – review and editing; methodology; project administration; supervision; formal analysis; resources; validation; funding acquisition; writing – original draft; investigation; visualization.

## CONFLICT OF INTEREST STATEMENT

The authors declare no conflicts of interest.

## Supporting information


**Data S1.** Supporting Information.

## Data Availability

The data that support the findings of this study are openly available in Figshare at https://doi.org/10.6084/m9.figshare.27158706.v1, reference number 10.6084/m9.figshare.27158706.v1.
