# Supplementary material for: Host‐feeding preferences of Culex pipiens and its potential significance for flavivirus transmission in the Camargue, France
Source: Med Vet Entomol. 2025 Mar 21;39(3):614–25. doi: 10.1111/mve.12802 (PMC12323745; doi:10.1111/mve.12802)
Supplement: Supplementary file 1 — Data S1. Supporting Information. [file MVE-39-614-s001.docx]

**Supplementary information**


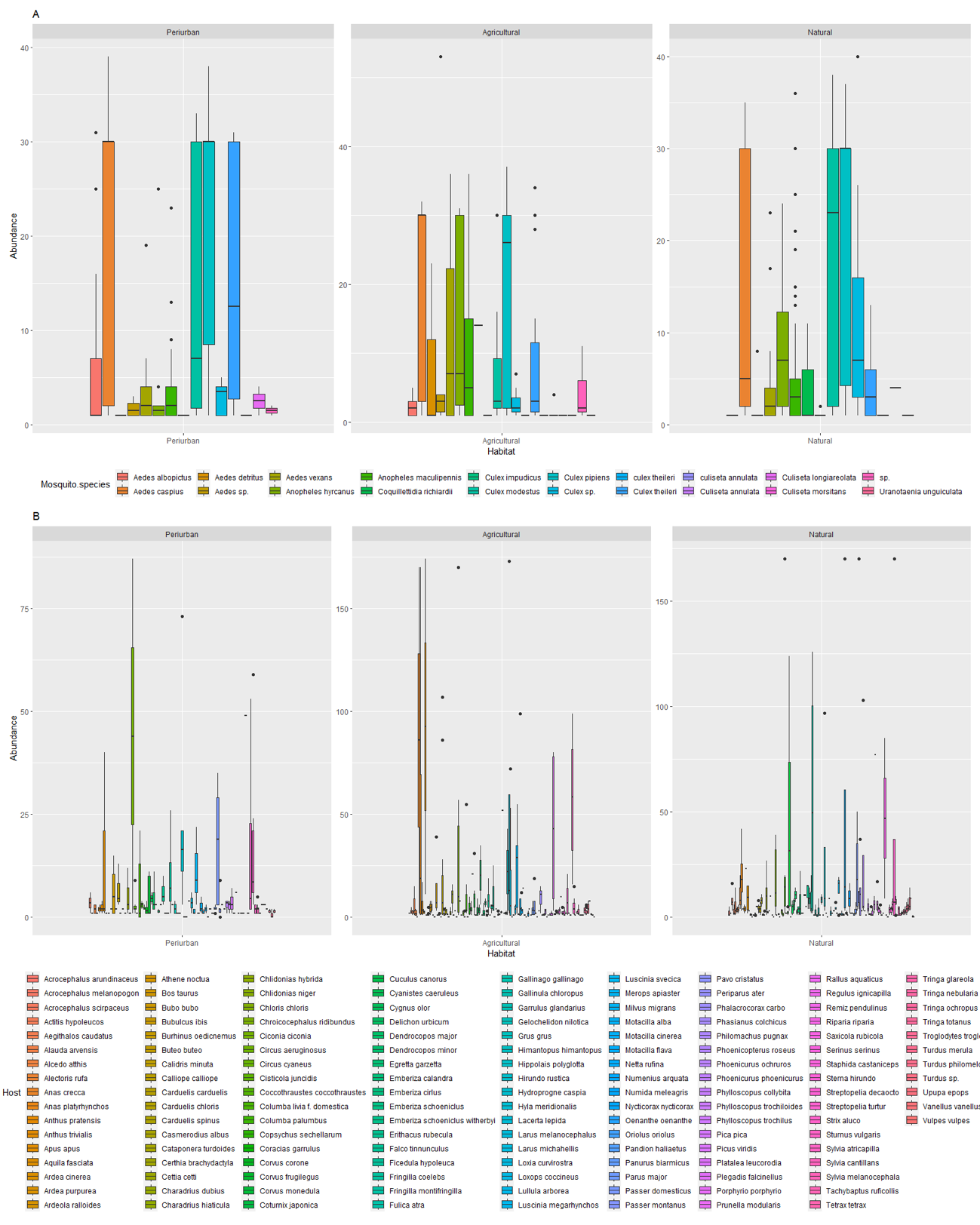
**Figure S1.** A) Mosquito abundances and B) Bird abundances per land use type (peri-urban, agricultural, natural) in the Camargue region. Each species is represented with a different colour in the graph (see legend).

**
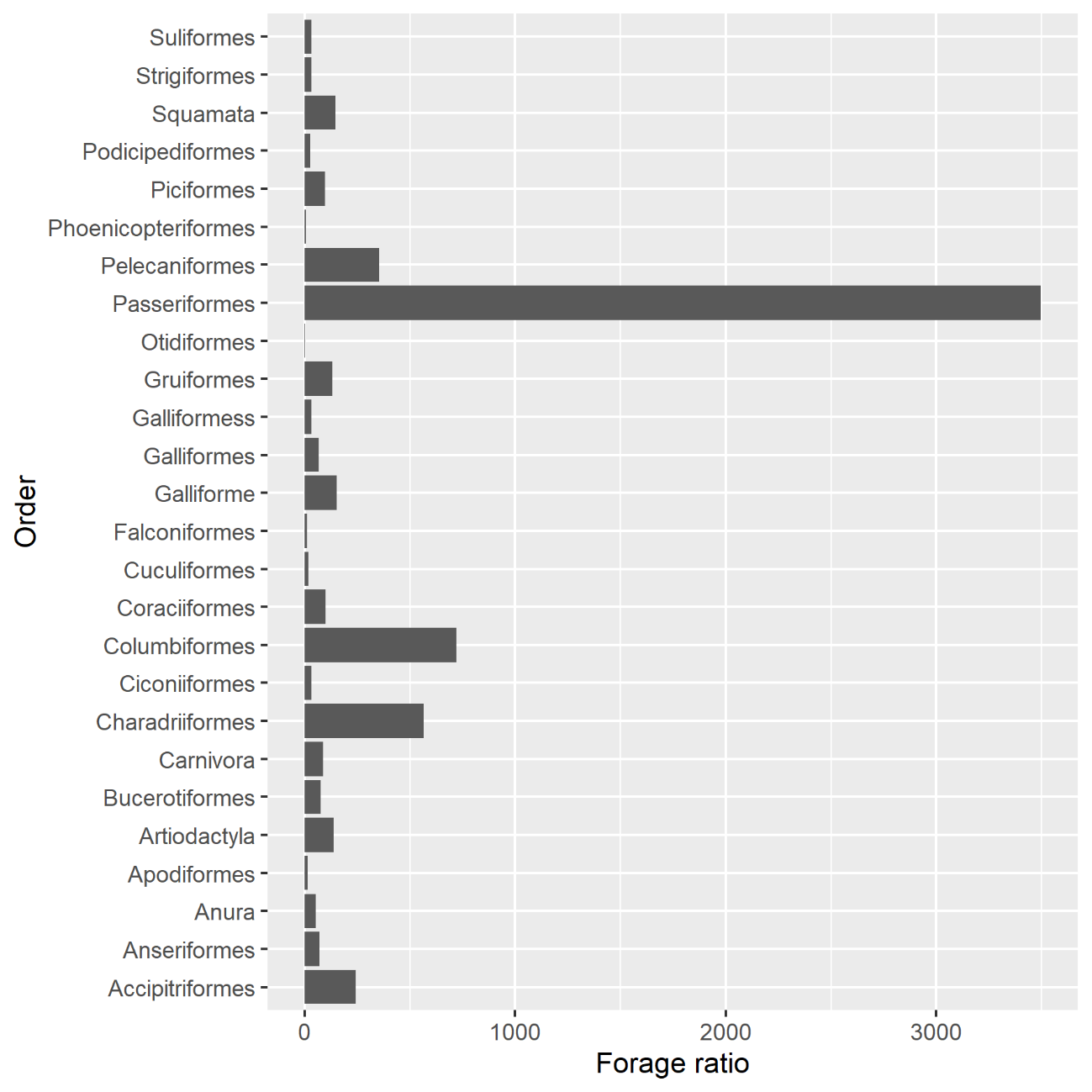
**

**Figure S2.** Accumulated forage ratios per vertebrate order.

**
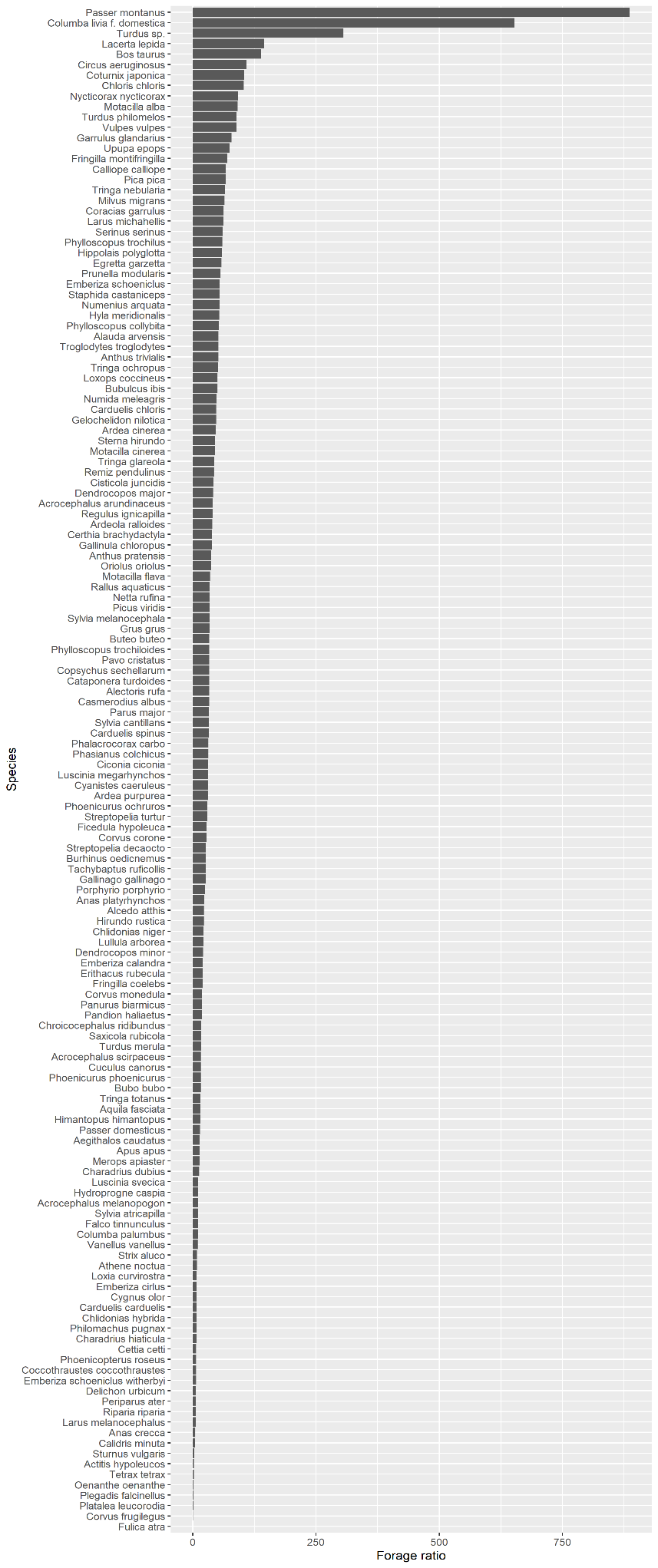
**

**Figure S3.** Forage ratios for the different host species in all localities combined (accumulated sum).

**
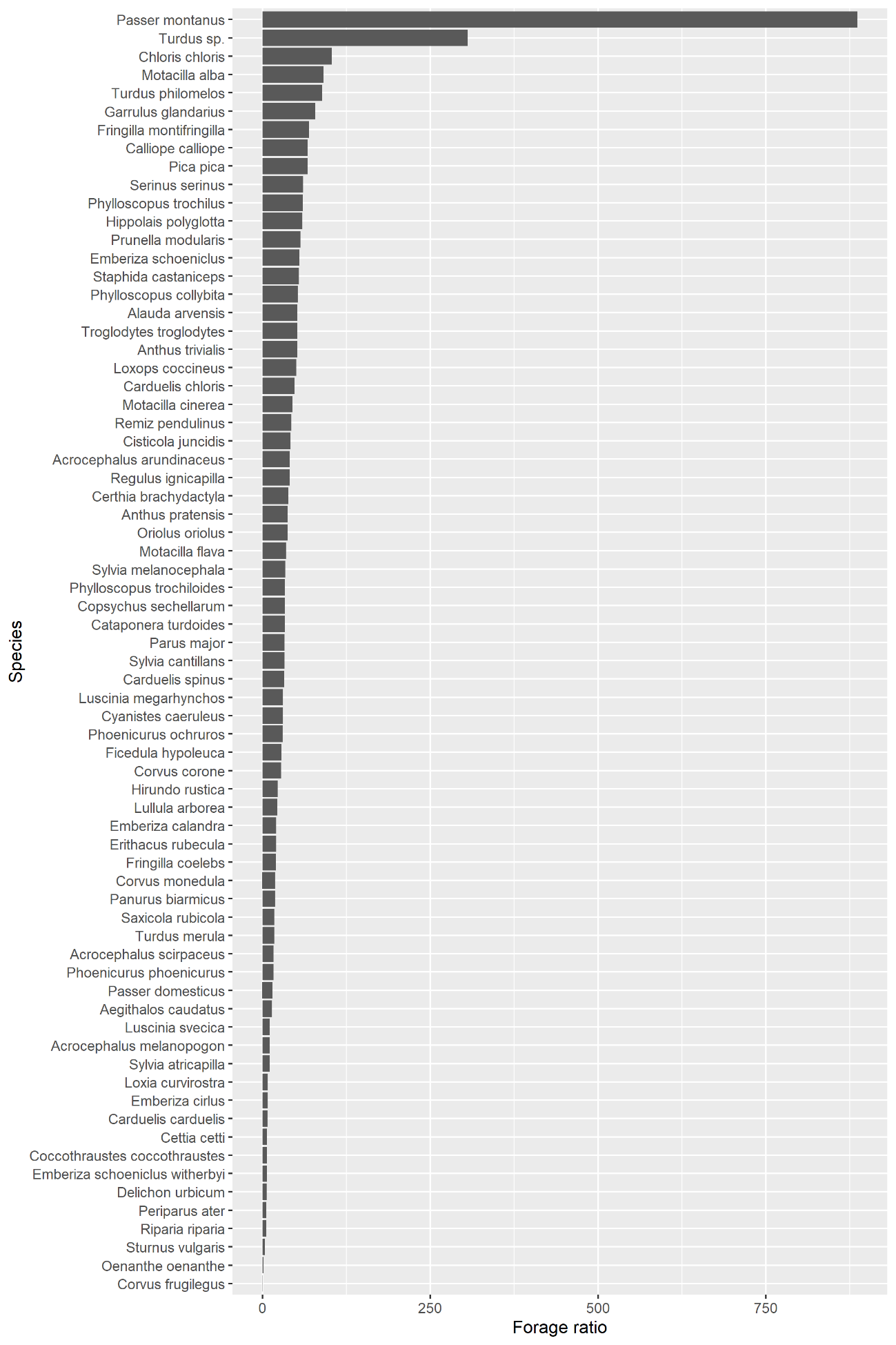
**

**Figure S4.** Forage ratios per bird species of Passeriformes.

**
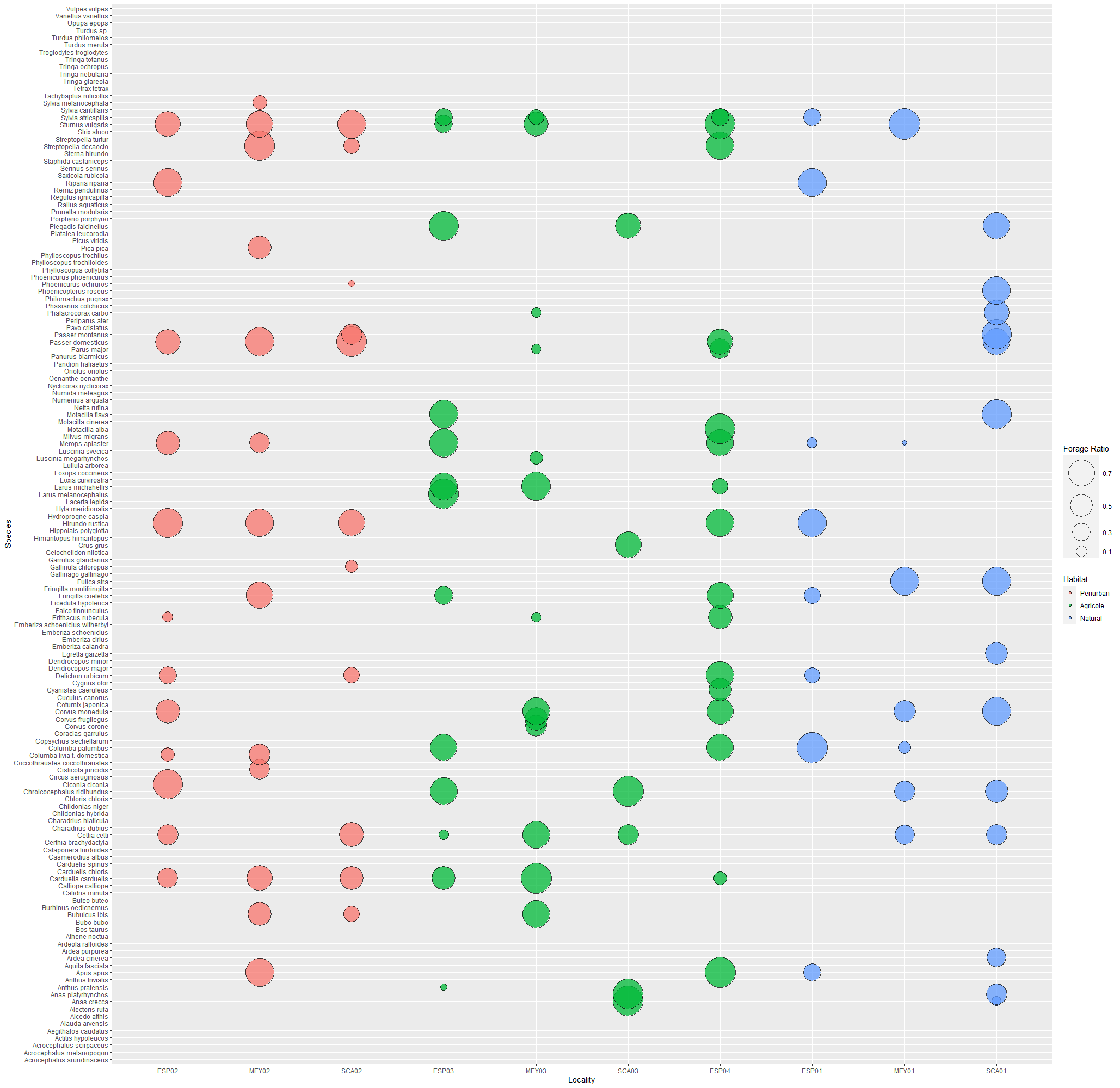
Figure S5.** Host avoidance based on the complementary of the 0-1 forage ratios of *Culex pipiens* for vertebrate hosts in the different localities and habitats: peri-urban (red), agricultural (green), natural (blue). Higher values mean greater avoidance and less preference for these host species.

**
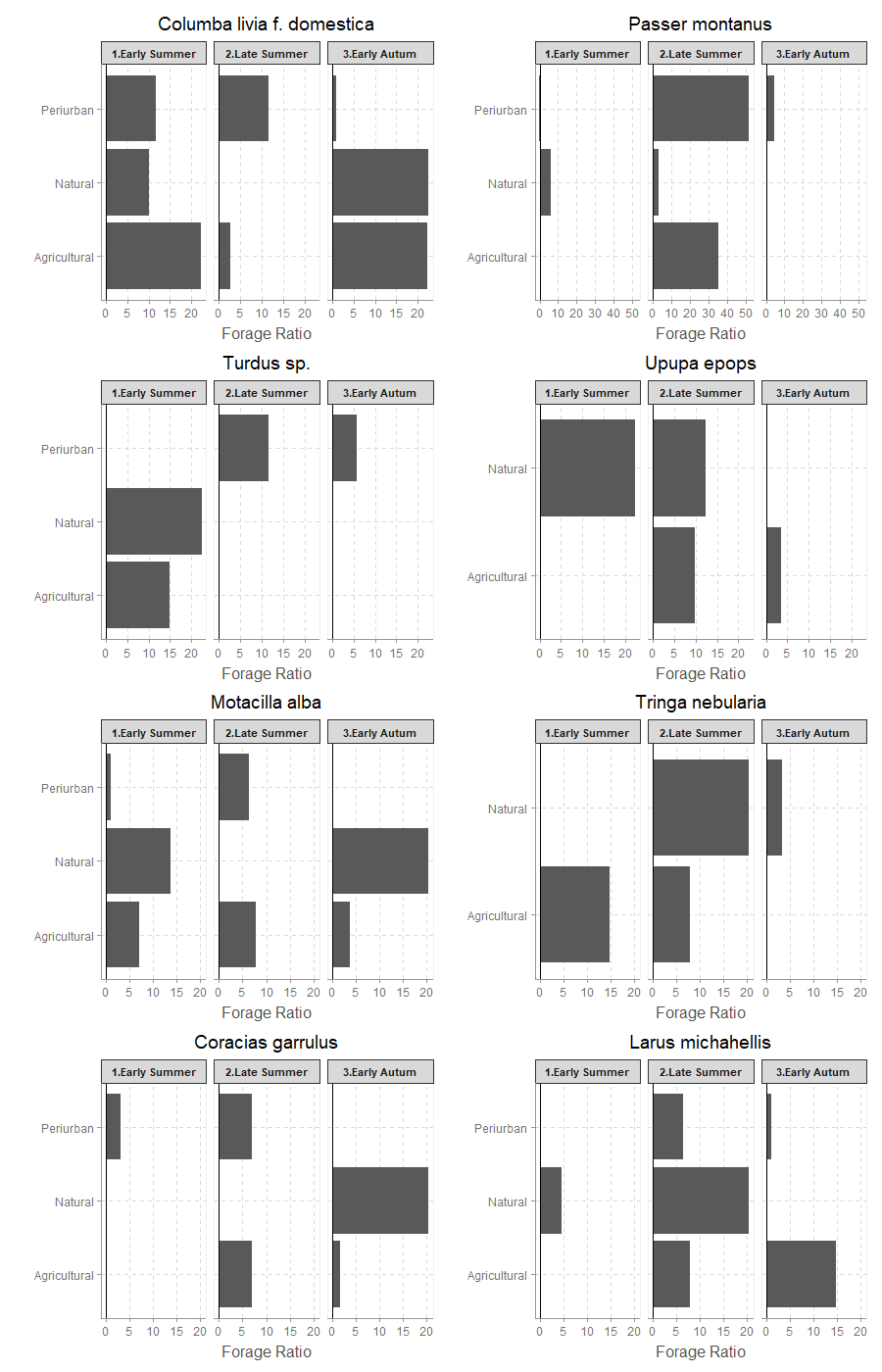
**

**Figure S6. Seasonal changes in the forage ratios of the species with higher selectivity values.** Censuses were carried out in early summer (July), late summer (August), and early autumn (October), and blood meal identification was aggregated for the weeks around these periods.

| 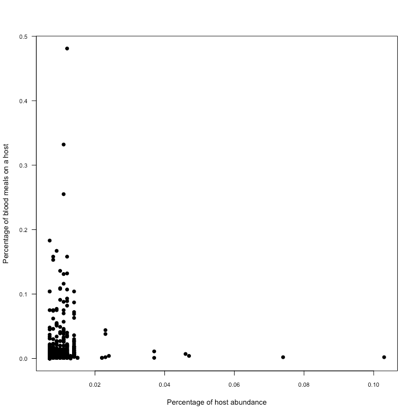 | 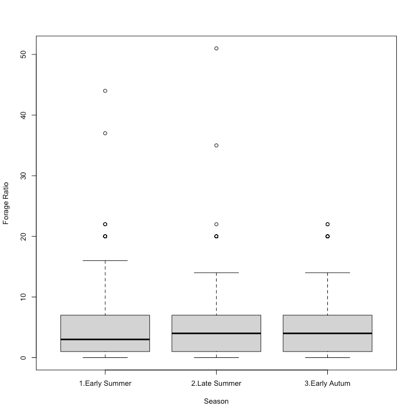 | 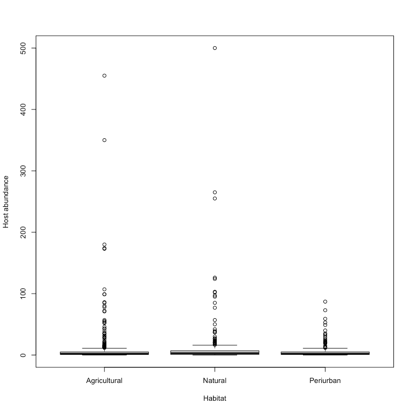 |
| --- | --- | --- |

**Figure S7.** Left panel: Relationship among host abundance (percentage) and blood meals on a host (percentage). Central panel: Relationship among season and forage ratio. Right panel: Relationship among habitat and host abundance.


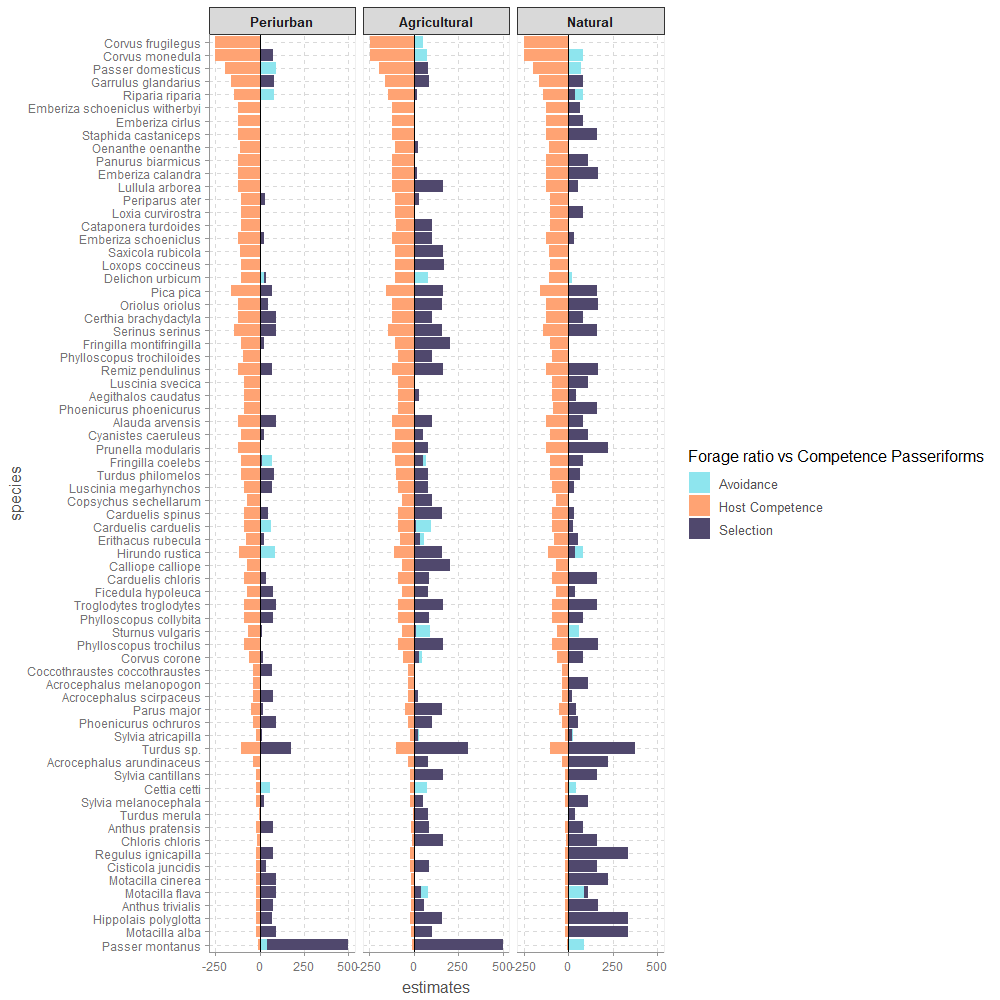
**Figure S8.** Forage ratios and potential transmission risk, based on host competence calculated from molecular prevalence per Passeriformes species.

| **Site** | **Habitat** | **Agricultural areas** | **Forest and bushes** | **Humid areas** | **Artificial surfaces** | **Water surface** |
| --- | --- | --- | --- | --- | --- | --- |
| **ESP01** | Natural | 26 | 36 | 38 | 0 | 0 |
| **ESP02** | Periurban | 47 | 0 | 0 | 53 | 0 |
| **ESP03** | Agricultural | 75 | 0 | 25 | 0 | 0 |
| **ESP03B** | Agricultural | 83 | 17 | 0 | 0 | 0 |
| **MEY01_1** | Natural | 16 | 0 | 29 | 0 | 55 |
| **MEY01-2** | Natural | 55 | 0 | 15 | 0 | 30 |
| **MEY02** | Periurban | 73 | 0 | 0 | 27 | 0 |
| **MEY03_2** | Agricultural | 27 | 0 | 73 | 0 | 0 |
| **SCA01_1** | Natural | 2 | 0 | 98 | 0 | 0 |
| **SCA01_2** | Natural | 6 | 0 | 94 | 0 | 0 |
| **SCA02_1** | Periurban | 18 | 0 | 67 | 15 | 0 |
| **SCA02_2** | Periurban | 33 | 0 | 53 | 14 | 0 |
| **SCA03_1** | Agricultural | 45 | 0 | 55 | 0 | 0 |
| **SCA03_2** | Agricultural | 42 | 0 | 58 | 0 | 0 |

**Table S1.** Characteristics of the landscapes (% each land cover) in a 600 m-radius buffer zone around the BG trap sites extracted from the CORINE land cover1 geographical database. SCA01 is the natural area of Scamandre, SCA02 the peri-urban port of Gallician and SCA03 is an agricultural area in Mas Quartret. ESP01 is the natural forest and marshes of Espeyran, ESP02 is the peri-urban area of Saint-Gilles, ESP03 is the agricultural area of the field of Espeyran being ESP03B the area of the Château d’Espeyran. MEY01 is the natural area of the Meyranne swamp, MEY02 is the peri-urban area of Mas Thibert and MEY03 is the agricultural area around Meyranne.

| **Mosquito species** | **ESP01** | **ESP02** | **ESP03** | **ESP03B** | **MEY01** | **MEY02** | **MEY03** | **SCA01** | **SCA02** | **SCA03** | **Total** |
| --- | --- | --- | --- | --- | --- | --- | --- | --- | --- | --- | --- |
| ***Aedes albopictus*** |  | 24 |  | 4 | 2 | 97 | 5 |  | 5 | 11 | **148** |
| ***Aedes caspius*** | 551 | 1053 | 1056 | 4598 | 513 | 2975 | 149 | 875 | 853 | 1689 | **14312** |
| ***Aedes detritus*** |  |  |  | 1 |  | 1 |  |  |  | 24 | **26** |
| ***Aedes sp.*** | 2 | 2 | 13 | 53 | 10 | 5 |  |  |  | 2 | **87** |
| ***Aedes vexans*** | 33 | 20 | 108 | 367 | 67 | 60 | 627 |  |  | 136 | **1418** |
| ***Anopheles hyrcanus*** | 57 | 28 | 44 | 28 | 42 | 8 | 10 | 27 | 4 | 172 | **420** |
| ***Anopheles maculipennis*** | 128 | 52 | 1458 | 1965 | 907 | 112 | 28 | 26 | 24 | 372 | **5072** |
| ***Coquillettidia richiardii*** |  |  |  |  | 13 |  |  |  |  | 14 | **27** |
| ***Culex impudicus*** |  |  |  | 3 | 9 | 2 |  | 2 |  |  | **16** |
| ***Culex modestus*** | 7 | 9 | 9 | 10 | 816 | 9 |  | 155 | 250 | 126 | **1391** |
| ***Culex pipiens*** | 602 | 2556 | 612 | 768 | 3686 | 868 | 526 | 2129 | 2257 | 1967 | **15971** |
| ***Culex sp.*** |  | 10 | 1 | 2 | 79 | 8 | 5 | 33 | 11 | 12 | **161** |
| ***Culex theileri*** | 24 | 26 | 71 | 98 | 15 | 251 | 9 | 33 | 16 | 15 | **558** |
| ***Culiseta annulata*** | 1 |  | 5 | 3 |  | 2 | 3 |  |  |  | **14** |
| ***Culiseta longiareolata*** |  | 5 |  | 1 |  |  |  |  |  |  | **6** |
| ***Culiseta morsitans*** |  |  |  |  |  |  |  |  |  | 1 | **1** |
| ***Uranotaenia unguiculata*** |  |  |  |  | 1 |  | 1 |  |  | 1 | **3** |
| **Total** | **1405** | **3785** | **3377** | **7901** | **6160** | **4398** | **1363** | **3280** | **3420** | **4542** | **39631** |
| **Habitat** | NAT | PURB | AGR | AGR | NAT | PURB | AGR | NAT | PURB | AGR |  |

**Table S2.** Results of the mosquito capture in all localities across all seasons. NAT: Natural areas, AGR: Agricultural areas and PURB: Peri-urban areas.

|  | **NAT** | **NAT** | **NAT** | **AGR** | **AGR** | **AGR** | **AGR** | **PURB** | **PURB** | **PURB** |  |
| --- | --- | --- | --- | --- | --- | --- | --- | --- | --- | --- | --- |
| **Host species** | **SCA01** | **MEY01** | **ESP01** | **ESP03B** | **MEY03** | **SCA03** | **ESP03** | **ESP02** | **MEY02** | **SCA02** | **Total** |
| ***Acrocephalus arundinaceus*** | 1 | 6 |  |  | 2 | 2 |  |  |  |  | **11** |
| ***Acrocephalus melanopogon*** | 2 |  |  |  |  |  |  |  |  |  | **2** |
| ***Acrocephalus scirpaceus*** | 10 | 23 |  |  | 4 | 8 |  |  | 1 | 6 | **52** |
| ***Actitis hypoleucos*** | 9 |  |  |  |  |  |  |  |  |  | **9** |
| ***Aegithalos caudatus*** | 5 | 27 | 5 | 3 | 4 |  |  |  |  |  | **44** |
| ***Alauda arvensis*** |  | 5 | 2 | 3 | 1 |  | 15 | 1 | 1 | 1 | **29** |
| ***Alcedo atthis*** | 8 | 15 |  |  | 7 | 4 | 2 | 4 |  | 4 | **44** |
| ***Anas crecca*** | 24 | 8 |  |  |  | 182 |  |  |  |  | **214** |
| ***Anas platyrhynchos*** | 38 | 14 |  |  | 2 | 57 |  | 1 |  | 1 | **113** |
| ***Anthus pratensis*** |  | 4 |  | 1 | 5 | 2 | 17 |  | 1 | 3 | **33** |
| ***Anthus trivialis*** |  | 2 | 6 | 2 | 2 | 4 | 3 | 2 | 1 | 4 | **26** |
| ***Apus apus*** |  |  | 23 | 174 |  |  | 11 | 2 | 40 | 1 | **251** |
| ***Aquila fasciata*** |  |  |  |  |  | 1 |  |  |  |  | **1** |
| ***Ardea cinerea*** | 34 | 2 |  | 1 | 3 | 7 | 2 |  |  | 1 | **50** |
| ***Ardea purpurea*** |  | 3 |  |  | 1 | 8 |  |  | 1 |  | **13** |
| ***Ardeola ralloides*** | 1 | 3 |  |  |  | 10 |  | 2 |  |  | **16** |
| ***Athene noctua*** |  |  |  | 1 |  |  |  |  |  |  | **1** |
| ***Bubo bubo*** |  |  | 1 |  |  |  |  |  |  |  | **1** |
| ***Bubulcus ibis*** | 4 | 2 | 1 |  | 42 |  | 14 | 1 | 16 | 9 | **89** |
| ***Burhinus oedicnemus*** |  |  |  |  | 1 |  | 1 |  |  |  | **2** |
| ***Buteo buteo*** |  |  |  | 2 | 3 | 1 | 3 | 2 |  |  | **11** |
| ***Calidris minuta*** | 5 |  |  |  |  |  |  |  |  |  | **5** |
| ***Carduelis carduelis*** |  | 12 | 16 | 10 | 198 | 15 | 36 | 15 | 21 | 15 | **338** |
| ***Carduelis chloris*** |  | 7 | 1 | 5 | 2 | 2 | 2 |  |  | 2 | **21** |
| ***Carduelis spinus*** |  | 11 | 5 |  | 5 | 1 |  | 2 |  | 2 | **26** |
| ***Casmerodius albus*** | 2 | 2 |  |  |  | 3 |  |  |  |  | **7** |
| ***Certhia brachydactyla*** |  | 5 | 2 | 8 | 1 |  | 5 | 1 |  |  | **22** |
| ***Cettia cetti*** | 39 | 54 | 10 |  | 39 | 28 | 18 | 16 | 5 | 17 | **226** |
| ***Charadrius dubius*** | 5 |  |  | 1 |  |  |  |  |  |  | **6** |
| ***Charadrius hiaticula*** | 3 |  |  |  |  |  |  |  |  |  | **3** |
| ***Chlidonias hybrida*** | 10 |  |  |  |  | 3 |  |  |  |  | **13** |
| ***Chlidonias niger*** | 1 |  |  |  |  |  |  |  |  |  | **1** |
| ***Chroicocephalus ridibundus*** | 46 | 59 |  | 1 | 8 | 90 | 67 |  |  | 1 | **272** |
| ***Ciconia ciconia*** | 1 |  |  |  | 8 |  |  | 87 | 1 |  | **97** |
| ***Circus aeruginosus*** | 3 |  |  |  | 1 | 1 |  |  |  |  | **5** |
| ***Cisticola juncidis*** |  | 7 | 1 |  | 3 | 5 | 2 | 5 | 12 | 2 | **37** |
| ***Coccothraustes coccothraustes*** |  |  |  |  |  |  |  |  |  | 1 | **1** |
| ***Columba livia f. domestica*** |  | 2 | 2 |  | 3 |  |  | 22 | 26 |  | **55** |
| ***Columba palumbus*** |  | 39 | 592 | 30 | 10 | 8 | 60 | 2 | 4 |  | **745** |
| ***Coracias garrulus*** |  | 2 |  | 11 | 2 |  | 6 | 2 | 3 | 2 | **28** |
| ***Corvus corone*** | 7 | 6 | 2 |  | 18 | 6 | 4 |  | 4 | 4 | **51** |
| ***Corvus frugilegus*** |  |  |  |  | 21 |  |  |  |  |  | **21** |
| ***Corvus monedula*** | 83 | 63 |  | 27 | 41 |  | 6 | 22 | 1 | 1 | **244** |
| ***Cuculus canorus*** |  |  |  |  |  |  | 1 |  |  |  | **1** |
| ***Cyanistes caeruleus*** | 2 | 15 | 13 | 18 | 2 | 6 | 4 | 6 |  | 3 | **69** |
| ***Cygnus olor*** | 16 | 8 |  |  |  | 7 |  |  |  |  | **31** |
| ***Delichon urbicum*** |  |  | 21 | 41 |  |  |  | 13 | 2 | 9 | **86** |
| ***Dendrocopos major*** |  | 7 | 3 | 2 | 1 |  | 1 |  |  |  | **14** |
| ***Dendrocopos minor*** |  | 13 | 1 |  |  |  |  |  |  | 3 | **17** |
| ***Egretta garzetta*** | 44 | 7 | 1 | 1 | 1 | 11 | 3 | 2 |  | 1 | **71** |
| ***Emberiza calandra*** |  | 2 |  |  | 5 |  | 9 |  |  |  | **16** |
| ***Emberiza cirlus*** |  |  | 2 |  |  |  |  |  |  |  | **2** |
| ***Emberiza schoeniclus*** | 10 | 11 |  |  |  | 2 | 3 |  | 3 |  | **29** |
| ***Emberiza schoeniclus witherbyi*** |  | 5 |  |  |  |  |  |  |  |  | **5** |
| ***Erithacus rubecula*** | 4 | 15 | 15 | 20 | 11 | 5 | 8 | 10 | 3 | 5 | **96** |
| ***Falco tinnunculus*** |  |  |  | 2 |  |  |  |  |  | 1 | **3** |
| ***Ficedula hypoleuca*** |  | 14 | 4 | 6 | 6 | 2 | 5 |  | 1 |  | **38** |
| ***Fringilla coelebs*** |  | 4 | 22 | 27 | 5 | 3 | 25 | 9 | 26 | 6 | **127** |
| ***Fringilla montifringilla*** |  |  |  |  |  |  |  |  | 3 |  | **3** |
| ***Fulica atra*** | 133 | 199 |  |  |  |  |  |  |  |  | **332** |
| ***Gallinago gallinago*** | 1 |  |  |  |  | 4 |  |  |  |  | **5** |
| ***Gallinula chloropus*** | 6 | 9 |  |  | 1 | 2 | 6 | 1 |  | 8 | **33** |
| ***Garrulus glandarius*** |  |  | 2 | 1 |  |  | 2 |  |  |  | **5** |
| ***Gelochelidon nilotica*** | 9 | 2 | 3 |  |  | 3 | 2 | 1 |  |  | **20** |
| ***Grus grus*** |  | 1 |  |  |  | 52 |  |  |  |  | **53** |
| ***Himantopus himantopus*** | 11 | 6 |  |  |  | 2 |  |  |  |  | **19** |
| ***Hippolais polyglotta*** |  | 1 |  |  | 3 | 1 |  |  |  | 1 | **6** |
| ***Hirundo rustica*** | 12 | 9 | 97 | 43 |  | 1 |  | 84 | 33 | 24 | **303** |
| ***Hydroprogne caspia*** | 2 |  |  |  |  |  |  |  |  |  | **2** |
| ***Larus melanocephalus*** |  |  | 9 | 6 |  |  | 195 | 4 |  |  | **214** |
| ***Larus michahellis*** | 10 | 1 | 1 | 11 | 73 | 2 | 66 | 7 |  |  | **171** |
| ***Loxia curvirostra*** |  |  | 2 |  |  |  |  |  |  |  | **2** |
| ***Lullula arborea*** |  |  | 3 |  |  |  | 1 |  |  |  | **4** |
| ***Luscinia megarhynchos*** |  | 11 | 8 | 3 | 12 | 2 | 9 | 3 | 3 | 1 | **52** |
| ***Luscinia svecica*** | 2 |  |  |  |  |  |  |  |  |  | **2** |
| ***Merops apiaster*** |  | 34 | 18 | 29 | 5 | 2 | 90 | 22 | 12 |  | **212** |
| ***Milvus migrans*** | 1 | 2 |  |  | 1 |  | 2 |  | 1 |  | **7** |
| ***Motacilla alba*** | 1 | 1 | 3 | 100 | 1 | 4 | 9 | 1 | 5 | 2 | **127** |
| ***Motacilla cinerea*** | 1 | 3 |  |  |  |  |  | 1 |  | 3 | **8** |
| ***Motacilla flava*** | 239 | 3 | 5 | 2 | 5 | 6 | 92 | 1 |  | 3 | **356** |
| ***Netta rufina*** | 1 | 3 |  |  |  | 14 |  |  |  |  | **18** |
| ***Numenius arquata*** |  | 1 |  |  | 2 | 1 |  |  |  |  | **4** |
| ***Nycticorax nycticorax*** | 96 |  |  |  | 79 |  |  |  |  |  | **175** |
| ***Oenanthe oenanthe*** |  |  |  | 4 |  |  |  |  |  |  | **4** |
| ***Oriolus oriolus*** |  | 2 |  |  |  | 1 |  | 2 |  |  | **5** |
| ***Pandion haliaetus*** | 2 |  |  |  |  |  |  |  | 1 |  | **3** |
| ***Panurus biarmicus*** | 3 | 3 |  |  |  |  |  |  |  |  | **6** |
| ***Parus major*** | 5 | 12 | 9 | 14 | 11 | 1 | 9 | 6 | 4 | 5 | **76** |
| ***Passer domesticus*** | 83 | 6 |  | 24 |  | 2 |  | 25 | 48 | 87 | **275** |
| ***Passer montanus*** | 158 | 12 | 4 |  |  |  |  | 4 |  | 12 | **190** |
| ***Periparus ater*** |  |  |  | 3 |  |  |  | 3 |  |  | **6** |
| ***Phalacrocorax carbo*** | 62 | 2 |  |  | 11 | 2 | 15 | 2 |  |  | **94** |
| ***Phasianus colchicus*** |  |  |  |  | 1 | 1 | 3 |  |  |  | **5** |
| ***Philomachus pugnax*** | 3 |  |  |  |  |  |  |  |  |  | **3** |
| ***Phoenicopterus roseus*** | 111 | 5 |  |  |  |  |  |  |  |  | **116** |
| ***Phoenicurus ochruros*** |  |  | 3 | 3 | 1 |  |  | 1 | 4 | 7 | **19** |
| ***Phoenicurus phoenicurus*** |  |  | 1 |  |  |  |  |  |  |  | **1** |
| ***Phylloscopus collybita*** | 4 | 9 | 2 | 1 | 4 | 4 | 2 | 4 | 1 | 3 | **34** |
| ***Phylloscopus trochilus*** |  | 2 | 1 |  | 1 |  | 1 |  |  |  | **5** |
| ***Pica pica*** | 2 | 8 | 1 | 4 | 2 |  | 1 | 8 | 16 | 1 | **43** |
| ***Picus viridis*** |  | 7 |  | 2 | 6 |  | 1 |  | 1 |  | **17** |
| ***Platalea leucorodia*** | 12 |  |  |  |  |  |  |  |  |  | **12** |
| ***Plegadis falcinellus*** | 77 |  |  |  | 9 | 46 | 158 |  |  |  | **290** |
| ***Porphyrio porphyrio*** | 14 | 29 |  |  | 2 | 1 |  |  |  | 6 | **52** |
| ***Prunella modularis*** | 1 | 4 | 5 | 2 | 2 | 2 | 3 |  |  |  | **19** |
| ***Rallus aquaticus*** | 5 | 11 |  |  | 1 | 8 | 2 |  |  | 1 | **28** |
| ***Regulus ignicapilla*** |  | 1 |  |  |  |  |  |  | 1 |  | **2** |
| ***Remiz pendulinus*** |  | 2 |  |  |  | 5 | 1 |  |  | 1 | **9** |
| ***Riparia riparia*** |  | 9 | 85 |  |  |  | 10 | 49 |  |  | **153** |
| ***Saxicola rubicola*** |  |  |  |  | 9 |  | 1 |  |  |  | **10** |
| ***Serinus serinus*** |  |  | 1 | 5 | 1 | 1 |  | 1 | 1 |  | **10** |
| ***Sterna hirundo*** | 6 | 1 |  |  |  | 2 |  |  |  |  | **9** |
| ***Streptopelia decaocto*** | 9 |  | 1 | 42 |  |  | 3 | 6 | 106 | 9 | **176** |
| ***Streptopelia turtur*** |  | 3 | 9 |  |  | 2 | 2 |  |  |  | **16** |
| ***Strix aluco*** |  |  |  | 1 |  |  |  |  |  |  | **1** |
| ***Sturnus vulgaris*** |  | 2019 | 11 | 104 | 25 |  | 24 | 27 | 27 | 40 | **2277** |
| ***Sylvia atricapilla*** |  | 17 | 23 | 12 | 13 | 7 | 23 | 7 | 5 | 6 | **113** |
| ***Sylvia cantillans*** |  |  | 1 |  |  |  | 1 |  |  |  | **2** |
| ***Sylvia melanocephala*** | 2 |  | 14 | 2 | 2 | 3 | 7 | 5 | 9 | 3 | **47** |
| ***Tachybaptus ruficollis*** | 2 | 4 |  |  |  |  |  |  |  | 1 | **7** |
| ***Tetrax tetrax*** |  |  |  |  |  |  |  |  |  | 3 | **3** |
| ***Tringa glareola*** | 2 | 4 |  | 2 | 6 | 1 |  |  |  | 3 | **18** |
| ***Tringa nebularia*** | 4 | 1 |  |  | 1 | 1 |  |  |  |  | **7** |
| ***Tringa ochropus*** | 8 | 3 |  | 1 | 1 |  | 1 | 4 |  |  | **18** |
| ***Tringa totanus*** |  |  |  |  |  | 1 |  |  |  |  | **1** |
| ***Troglodytes troglodytes*** |  | 7 | 1 |  | 6 |  | 1 | 1 | 2 |  | **18** |
| ***Turdus merula*** |  | 9 | 11 | 8 | 9 | 2 | 8 |  |  |  | **47** |
| ***Turdus philomelos*** | 14 | 5 | 3 | 8 | 2 | 2 | 6 | 2 |  |  | **42** |
| ***Upupa epops*** |  |  | 1 | 2 |  |  | 1 |  |  |  | **4** |
| ***Vanellus vanellus*** |  |  |  |  | 1 |  |  |  |  |  | **1** |
| **Total** | **1531** | **2967** | **1395** | **836** | **996** | **675** | **1091** | **695** | **496** | **539** | **11221** |

**Table S3.** Results of the bird censuses in all localities across all seasons. NAT: Natural areas, AGR: Agricultural areas, PURB: Peri-urban area

| **Dependent and independent**  **variables** | **Values** | **Estimate** | **Standard error** | **Z value** | **p-value** |
| --- | --- | --- | --- | --- | --- |
| **Percentage of blood meals on a host vs host abundance** | Intercept | -4.60 | 0.33 | -13.78 | <0.0001 |
|  | Percentage of host abundance | 0.52 | 10.56 | 0.05 | 0.96 |
| **Forage Ratio**  **vs Season** | Early Summer vs Late summer | -0.07 | 0.08 | -0.90 | 0.6386 |
|  | Early summer vs Early autumn | -0.11 | 0.08 | -1.41 | 0.3317 |
|  | Late summer vs Early autumn | -0.04 | 0.08 | -0.52 | 0.8607 |
| **Host abundance**  **vs Habitat** | Agricultural vs Natural | -0.05 | 0.10 | -0.53 | 0.8551 |
|  | Agricultural vs Periurban | 0.54 | 0.11 | 4.64 | **<0.0001** |
|  | Natural vs Periurban | 0.59 | 0.12 | 4.96 | **<0.0001** |

**Table S4.** Model estimates for the GLM negative binomial among the percentage of blood meals on a host and percentage of host abundance. Model estimates including a post-hoc contrast test (as the response variable is a categorical variable) for the effect of season on forage ratio and the effect of habitat on host abundance. Related graphs are in supplementary material (Fig. S7).
